# Test anxiety and its determinants among health sciences students at Mattu University: a cross-sectional study

**DOI:** 10.3389/fpsyt.2023.1241940

**Published:** 2024-01-10

**Authors:** Mohammedamin Hajure Jarso, Mandaras Tariku, Aman Mamo, Tesfaye Tsegaye, Wubishet Gezimu

**Affiliations:** ^1^Department of Psychiatry, College of Health Sciences, Madda Walabu University, Shashemene, Ethiopia; ^2^Department of Psychiatry, College of Health and Medical Sciences, Hararmaya University, Harar, Ethiopia; ^3^Department of Nursing, College of Health Sciences, Madda Walabu University, Shashemene, Ethiopia; ^4^Department of Pharmacy, College of Health Sciences, Mattu University, Mattu, Ethiopia; ^5^Department of Nursing, College of Health Sciences, Mattu University, Mattu, Ethiopia

**Keywords:** test anxiety, determinants, health sciences students, Mattu University, Ethiopia

## Abstract

**Background:**

Test anxiety is a particular type of anxiety that is marked by physical, cognitive, and behavioural symptoms when taking and performing tests. It is defined as “severe stress” before, during, and after exams and other assessments. Test anxiety could cause poor academic performance and increase dropout rates. This study aimed to determine the levels of test anxiety and its determinants among health sciences students at Mattu University.

**Methods:**

An institution-based cross-sectional study was conducted among 421 selected students from June 1 to June 30, 2021. The study utilized the Westside Test Anxiety, the Oslo Social Support Scale, the Rosenberg Self-esteem Scale, and the Kessler Scale to assess test anxiety, social support, self-esteem, and psychological distress, respectively. The collected data were entered into EpiData version 3.1 and then exported to STATA version 14.0 for analysis. A linear regression model was used to determine factors associated with test anxiety. The multiple regression assumptions were checked for each variable. Statistically significant effects were assumed for a *p*-value of less than 0.05 at a 95% confidence interval in the multiple linear regression analysis.

**Results:**

A total of 416 (99%) participants were completed out of the 420 questionnaires administered. The mean score of test anxiety among participants was 25.3 (SD: ±5.51). Tobacco use (β: 1.028; 95% CI: 0.709–1.347), khat chewing (β: 0.115; 95% CI: 0.038–0.192), self-esteem (β: −0.049; 95% CI: −0.062–(−0.036)), psychological distress (β: 0.022; 95% CI: 0.017–0.027), and physical activity (β: -0.162; 95% CI: −0.224–(−0.099)) were shown to have a significant association with test anxiety.

**Conclusion:**

Test anxiety was common in the study area. Current substance use (tobacco use and khat chewing) and psychological distress were discovered to be factors that exacerbated test anxiety, whereas self-esteem and physical activity were discovered to be factors that alleviated test anxiety. Therefore, students and stakeholders need to work to discourage those factors that increase test anxiety while promoting factors that alleviate it.

## Introduction

Naturally, a little tension before a test is normal. However, excessive and persistent anxiety is detrimental and could impair a student’s academic performance. Test anxiety (TA) is defined as a psychological disorder in which a person feels stress at the time of, before, or after a test or other evaluation to the point where it adversely affects his or her ability or performance (1).

The etiology of TA is not attributed to a single agent. Individual, parent, and teacher characteristics, the student learning environment, and the nature of the education program account for the majority of the causative agents (2). Regarding the learning environment and nature of the program, medical and health sciences students are more exposed to TA than others due to the large credit hours and stressful clinical settings (3, 4).

Clinically, the student might experience some emotional, physical, and behavioural syndromes. These syndromes are related to the stimulation of the sympathetic nervous system. The emotional responses could be manifested by feelings of confusion, crying easily, feeling impossibility, fearfulness, terror, and frustration (1, 2). Physical signs can include, but are not limited to, agitation, rapid heartbeat, perspiration, headache, nausea, diarrhea, fainting spells, and nibbling on pens or nail covers. Poor focus, feeling drowsy, fidgeting, and a lack of confidence are some behavioural symptoms that a student may display when confronting the TA (2).

TA significantly impedes the student’s ability to accomplish his or her own tasks. It also endangers the student’s social, emotional, and behavioural development and feelings about himself and the learning environment (5). It is the primary cause of poor academic performance and increased attrition at the university (6). Besides its impact on academic achievement, extreme anxiety could lead to a mental disorder (7, 8).

Globally, the magnitude of TA has been erratically distributed among different health sciences students of different disciplines, like nursing, medical students, and others. For instance, according to a study conducted in the United States of America (USA), 55% of nursing students have experienced TA (9). A cohort study conducted at Arab Umm Al-Qura University showed that about 53.04% of medical students developed TA (10). Another study conducted in Malaysia among medical students revealed that 52% experienced TA (11). In India, about 85% of undergraduate health students experienced TA (12). In Ethiopia, two cross-sectional studies conducted at Addis Ababa University and the University of Gondar revealed a 52.30 and 54.7% prevalence of TA, respectively (13, 14).

According to scientific evidence, the students’ socio-demographic characteristics, psychological, social, and substance use traits were found to be factors determining TA. Cumulative grade point average (CGPA), age, year of study, family educational status, and department were socio-demographic variables determining TA (13–15). A study conducted at British University in Malaysia found that there was a higher level of TA score among female students compared to male students. The same study revealed that there was no independent association between TA and the student’s academic performance (16). Psychological determinants of test anxiety include, psychological distress, lower self-efficacy, preparedness, confidence in taking examinations, chronic stress, and low self-esteem (13–15, 17–20). Regarding social factors, poor social support and low socio-economic status were evidenced to determine TA (13, 20). Furthermore, in a study conducted at the University of Gondar, drinking alcohol (ever) was linked to TA (14).

In Ethiopia, however, little study has been conducted on TA among health science students. Hence, the aim of this study was to assess the levels of TA and identify its determinants among health sciences students at Mattu University. To our knowledge, this was the first study in Ethiopia that identified a linear relationship between TA and explanatory variables among health sciences students. The study was grounded on a null hypothesis: there is no significant association between explanatory variables and TA among health sciences students at Mattu University.

## Methods and materials

### Study design, period, and setting

**Figure 1:**
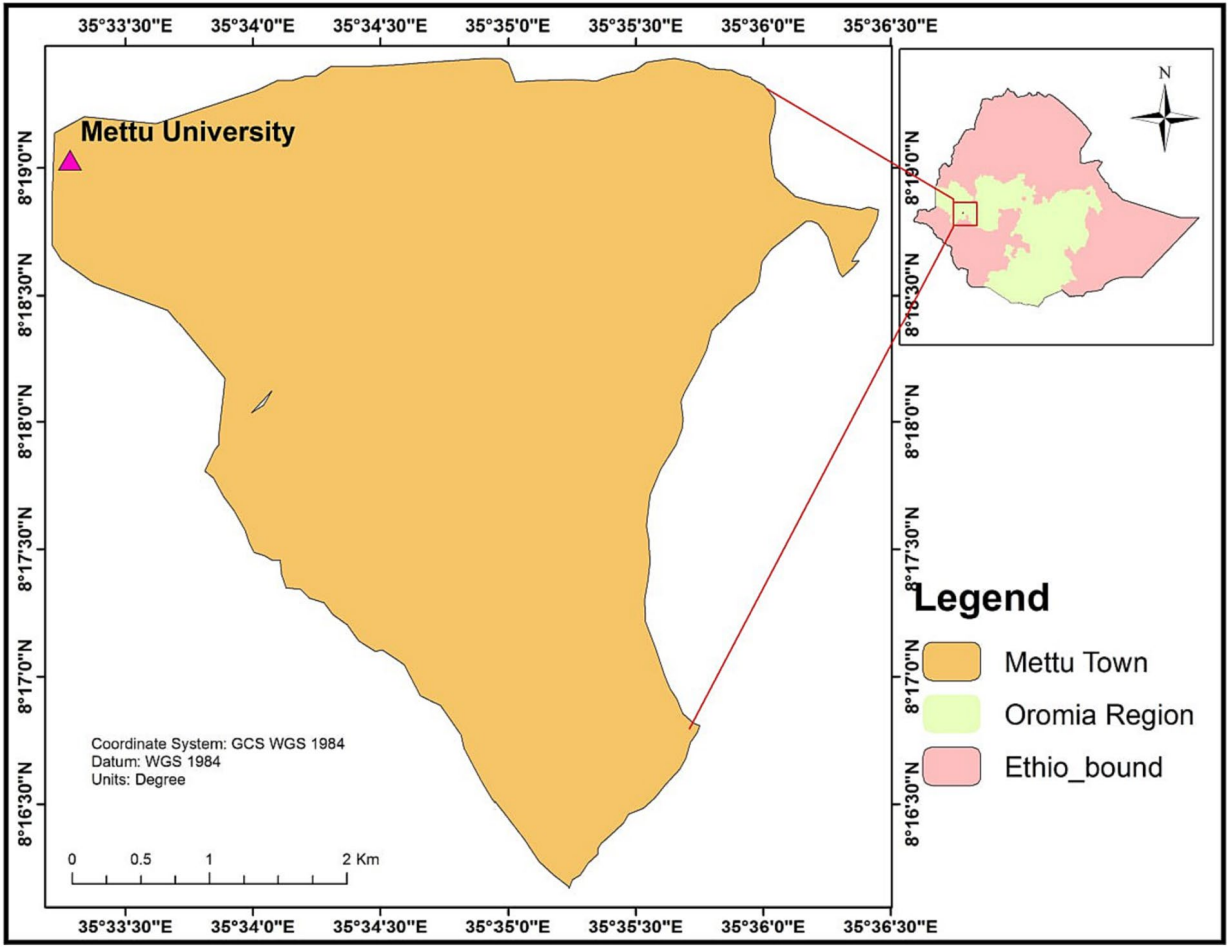
Geographical location of the study area.

An institution-based, cross-sectional study was conducted from June 1 to 30, 2021, in the College of Health Sciences, Mattu University. Mattu University is one of the nine Ethiopian higher public educational institutions established in 2011. It is located in Mettu town, in the evergreen environment of the Ilu Abba Bor zone, 600 km from Addis Ababa (Finfine), the capital of Ethiopia (Figure 1). The university is divided into two campuses: the main campus and the Bedelle campus. The college was enrolling students in six (6) departments during the study year (psychiatry nursing, health informatics, midwifery, nursing, health officers, and pharmacy). The Health Sciences College is located on the main campus of the university.

### Population selection and eligibility criteria

All undergraduate health science students at Mattu University were considered the source population, whereas all selected undergraduates at the college were study populations. All selected regular undergraduate students from the second to the fifth who were willing to participate were included in the study. Students enrolled in programs other than regular undergraduates were, however, excluded from this study. In addition, students who were severely ill during the survey period and who were not willing to participate were not included in this study.

### Sample size determination and sampling procedure

The sample size required for this study was determined by using the formula to estimate the single population means considering the following assumptions: a standard deviation (SD) of the mean test anxiety score of 9.75 (19), a confidence interval (95% CI) of 1.96 (Zα/2 = 1.96), a 1% margin of error (d, 0.01), and a non-response rate of 15%. Accordingly, the final sample size, *n* = 420, was used for this study.

First, the population was stratified based on their year of study (batches). Then the sample was proportionally allocated to each stratum. Finally, the study units were randomly selected using a lottery method (computer-generated). The total number of students in the college, along with their identification number, was used as a sampling framework.

### Outcome interest and study variables

TA was the outcome of interest in this study. The socio-demographic variables (age, sex, academic year (batch), monthly pocket money, family marital status, family size, and last semester GPA), psychosocial factors (self-esteem, psychological distress, and social support), substance use (tobacco use, khat chewing, and alcohol drinking), history of known chronic physical illness, negative life events, and physical activity were correlates (explanatory variables) tested over TA.

### Operational definitions

**Academic achievement:** the academic achievement of students as indicated by averaging the scores obtained from different subjects in the previous semester or grade point average (GPA).
**Current substance use** refers to participants who have used substances in the last 3 months prior to the survey.
**Chronic physical illness:** participants who had confirmed medical illnesses by health professionals like diabetes mellitus (DM), epilepsy, hypertension (HTN), and human immune virus/acquired immune deficiency syndrome (HIV/AIDS).

### Data collection instrument and techniques

Four instructors collected data using a pretested and standardized self-administrated questionnaire adapted from Westside Test Anxiety (WTAI). In addition, the questionnaire includes socio-demographic, clinical, and other psychological and behavioural-related questions. The WTAI is a self-announced questionnaire of 10 articulations on which respondents are requested to report how regularly they experience uneasiness manifestations prior to, during, and subsequent to stepping through exams. Every assertion reaction is scored on a 5-point Likert scale (1–5), yielding an absolute test nervousness score ranging from 10 to 50. Numbers were ascribed to five unique degrees of test nervousness as per the WTAI score: a score of 1.0–1.9 for serenely low-test nervousness; a score between 2.0–2.5 for ordinary or normal test nervousness; a score between 2.5–2.9 for high typical test tension; a score between 3.0–3.4 for moderately high; a score between 3.5–3.9 for high test nervousness; a score between 4.0–5.0 for extremely high tension; and the cut point for hazardous test uneasiness is 30. The Cronbach alpha was an alpha of 0.78, with a split-half unwavering quality of 0.77 in a Nigerian sample (21).

**Oslo social support scale**: It has a 3-item questionnaire commonly used to assess social support, and it was used in several studies, with the sum score scale ranging from 3–14, which is three broad categories: poor support (3–8), moderate support (9–11), and strong support (12–14) (22).
**Rosenberg Self-esteem Scale (RSES):** It was used to measure levels of self-esteem among the study participants. Students were scored based on a 4-point Likert scale, scoring “strongly disagree” as 1 point, “disagree” as 2 points, “agree” as 3 points, and “strongly agree” as 4 points. The sums of the scores for all ten items are kept on a continuous scale. Higher scores indicate higher self-esteem. The scale ranges from 0 to 30. Scores between 15 and 25 are within the normal range; scores below 15 suggest low self-esteem. Internal validity was 0.78–0.84, and reliability was 0.94, which is high (23).
**Kessler Scale (K10):** Psychological distress was measured using the Kessler Psychological Distress Scale (K-10). It is a simple measure of psychological distress; the K10 scale involves 10 questions about emotional states, each with a five-level response scale (24).

### Data quality control

Quality assurance measures were embedded in all aspects of the surveillance process. In the event that inconsistent or missing data were detected at any step during the data collection process, the questionnaire was returned to the data collectors for checks and corrections. The study used standardized study tools. Data cleaning and adjustments were conducted to avoid errors in the labeling or ordering of the variables of interest. Moreover, training was given to data collectors before the study’s commencement.

### Data processing and analysis

After collection, the data was edited and cleaned; each questionnaire was checked for completeness and coded. EpiData version 3.1 was used to enter data into a computer, and STATA version 14.0 was used for analysis. The categorical variable was described using frequency and percentage. Descriptive statistics and summary statistics were presented using text and tables. We tested for linearity, normality of the distributional normalcy, and multi-collinearity assumptions. A linear regression model was used to determine factors associated with test anxiety. In the multiple linear regression analysis, all independent variables with a *p*-value less than 0.25 in the bivariable analysis were included. The level of statistical significance was fixed at a *p*-value < 0.05. To assess the strength of statistical significance, unstandardized beta (β) coefficients with a 95% CI were used.

## Results

### Socio-demographic characteristics of participants

A total of 416 students participated in this study, which gives a response rate of 99%. More than half of the participants were male. The majority of participants, 362 (87%), were fourth-year students. Around two-thirds, or 272 (65.4%) of participants’ last semester grade point average (GPA), was between 2.76 and 3.75. Nearly half (49%) of participants earned about 500 to 1,000 Ethiopian Birr (ETB), where 1 ETB was equivalent to $0.0231 US, in pocket money monthly (Table 1).

**Table 1:** Socio-demographic characteristics of the health sciences students at Mattu University, 2021 (*n* = 416).

Variables	Categories	Frequencies	Percentages
Age	>20 year	108	26.0
	20–24 year	176	42.3
	24–27 year	132	31.7
Sex	Male	248	59.6
	Female	168	40.4
Academic year	Third year	54	13.0
	Fourth	362	87.0
Monthly pocket money	Less than 500 ETB	64	
	500–1,000 ETB	204	49.0
	Greater than 1,000 ETB	148	35.6
Family marital status	Never married	16	3.8
	Married	348	83.7
	Divorced	40	9.6
	Widowed	12	2.9
Family size	Living alone	16	3.8
	Two	16	3.8
	Three	64	15.4
	Four and above	320	77.0
Last semester GPA	2.00–2.75	48	11.5
	2.76–3.75	272	65.4
	3.76–4.00	96	23.1

GPA, grade point average; ETB, Ethiopian Birr.

### Levels of test anxiety score

**Figure 2:**
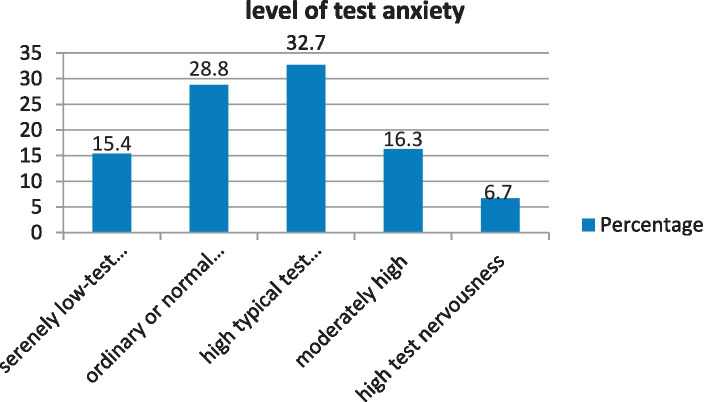
Levels of test anxiety among health sciences students at Mattu University.

In the current study, the mean score of TA among participants was 25.3 ± 5.51. The minimum and maximum scores of the WTAI were 13 and 38, respectively. The WTAI scale demonstrated a high internal consistency reliability coefficient (Cronbach’s alpha = 0:0.70). According to the WTAI score-specified degrees of test nervousness, nearly one-third (32.7%) of the participants experienced high typical test tension in the current study. A small proportion (6.7%) of participants experienced high test nervousness (Figure 2).

#### Psychosocial status of participants

The mean totals of self-esteem, social support, and psychological distress among the participants were 22.14 (SD: ±3.65), 8.85 (SD: ± 1.96), and 21.42 (SD: ±5.25), respectively (Table 2). The Kessler (psychological distress) scale revealed a high internal consistency reliability coefficient (Cronbach’s alpha = 0:0.72).

**Table 2:** Psychosocial status of health sciences students at Mattu University (*n* = 416).

Variables	Mean (±SD)	Range	
		Minimum	Maximum
Rosenberg (self-esteem)	22.14 ± 3.65	15	31
Social support	8.85 ± 1.96	4	14
Psychological distress	21.42 ± 5.25	10	36

SD, standard deviation.

#### Substance use, chronic physical illness, negative life event, and physical activity status of participants

The majority (87.5%) of participants reported that they have no chronic physical illnesses. About more than half (54%) of them did not engage in physical activity. Of the 160 participants who used substances in the last 3 months prior to the survey, 84 (52.5%), 72 (45%), and 4 (2.5%) drank alcohol, chewed khat, and used tobacco, respectively. Moreover, about two-thirds (66.3%) of participants had experienced a negative life event in the 6 months prior to the survey (Table 3).

**Table 3:** Substance use, chronic physical illness, negative life event, and physical activity status of participants of health sciences students at Mattu University (*n* = 416).

Variables	Category	Frequency	Percentage
Substance use (*n* = 160)	Tobacco	4	2.5
	Khat	72	45
	Alcohol	84	52.5
Chronic physical illness	No	364	87.5
	Yes	52	12.5
Physical activity	No	227	54.6
	Yes	189	45.4
Negative life event	No	140	33.7
	Yes	276	66.3

### Determinants of test anxiety

Table 4 shows the bivariate linear relationship between TA and independent variables. Accordingly, variables, including the age of participants, academic year, monthly pocket money, current substance use, family marital status, family size, last semester GPA, self-esteem, psychological distress, physical activity, and a negative life event in the last 6 months, were shown to be associated at a *p*-value less than 0.25 with a 95% CI.

**Table 4:** Bivariate linear regression analysis of the determinants of TA among health sciences students at Mattu University, 2021 (*n* = 416).

Variable	*R*2	*β*	Confidence interval (95%) for *β*		*p* value
			Lower	Upper	
Age	0.028	−0.085	−0.134	−0.037	<0.01
Gender (Female)	0.001	−0.027	−0.103	0.049	0.487
Academic year					
2nd	0.001	−0.183	−0.945	0.579	0.637
3rd	0.012	−0.125	−0.235	−0.014	0.027
4th	0.000	−0.001	−0.168	0.166	0.991
5th		Reference			
Monthly pocket money					
<500 ETB		Reference			
500–1,000 ETB	0.004	−0.051	−0.125	0.024	0.182
>1,000 ETB	0.008	−0.074	−0.151	0.004	0.062
Social support					
Poor	0.003	−0.040	−0.034	0.115	0.288
Moderate	0.000	0.016	−0.059	0.091	0.674
Strong		Reference			
Current use of substance					
Tobacco	0.043	0.825	0.451	1.19	<0.001
Khat	0.032	0.182	0.085	0.279	<0.001
Alcohol	0.007	−0.103	−0.224	0.018	0.342
Hx of chronic medical illness					
Yes	0.002	0.055	−0.058	0.168	0.339
No		Reference			
Family marital status					
Divorced	0.024	−0.202	−0.327	−0.077	0.002
Married	0.032	0.209	0.098	0.320	<0.001
Widowed		Reference			
Family size					
<3 members		Reference			
≥4 members	0.020	0.129	−0.205	0.114	0.004
Last semester GPA					
2.00–2.75		Reference			
2.76–3.75	0.035	0.152	0.075	0.229	<0.001
3.76–4.00	0.020	0.520	−0.217	−0.041	0.004
Self-esteem	0.057	−0.051	−0.071	−0.031	<0.001
Psychological distress	0.010	0.019	−0.190	0.121	0.003
Physical activity	0.079	−0.218	−0.290	−0.147	<0.001
Negative life event	0.010	0.082	0.003	0.160	0.042

Hx., history.

Explanatory variables in the final model described TA variation by about 42.3%. Multiple linear regression analysis output revealed that there was a significant association between tobacco use (β: 1.028; 95% CI: 0.709–1.347), khat chewing (β: 0.115; 95% CI: 0.038–0.192), self-esteem (β: -0.049; 95% CI: −0.062–(−0.036)), psychological distress (β: 0.022; 95% CI: 0.017–0.027), and physical activity (β: -0.162; 95% CI: −0.224–(−0.099)) and TA scores.

The mean score of TA was increased by the student’s current substance use, such as tobacco and khat chewing, by about 1.03 (*p* = ≤0.001) and 0.12 (*p* = 0.003), respectively. With an increase in a unit of self-esteem, the mean score of test anxiety decreased by 0.05 (*p* = ≤0.001). A one-unit increase in psychological distress increased the mean score of TA by 0.02 (*p* = ≤0.001). In addition, the TA of students who performed physical activity decreased by 0.16 units (*p* = ≤0.001) compared to those who did not perform physical activity (Table 5).

**Table 5:** Multiple linear regression analysis of the determinants of TA among health sciences students at Mattu University, 2021 (*n* = 416).

Variables	Unstandardized coefficients	*p*-value	Confidence interval (95%) for β	
			Lower boundary	Upper boundary
Constant	1.287	≤0.000	0.819	1.754
Age	−0.008	0.175	−0.019	0.003
Tobacco use	1.028	**≤0.001**	0.709	1.347
Khat chewing	0.115	**0.003**	0.038	0.192
Poor social support	−0.018	0.554	−0.077	0.042
Family size	0.031	0.382	−0.039	0.101
Year of study (third year)	−0.057	0.245	−0.154	0.039
Psychosocial distress	0.022	**≤0.001**	0.017	0.027
Physical activity	−0.162	**≤0.001**	−0.224	−0.099
Negative life events	−0.007	0.838	−0.070	0.057
Self-esteem	−0.049	**≤0.001**	−0.062	−0.036

Model summary: *R* = 65.1%, *R*^2^ = 42.3%, *p* < 0.001. *, statistical significance at *p*-value < 0.05; **, statistical significance at *p*-value < 0.001.

## Discussion

The present study assessed the levels of TA and its determinants among Mattu University students. Accordingly, the mean total test anxiety score using the WTAI was 25.3 ± 5.51. This finding is lower than a previous finding from Saudi Arabia, where the mean test anxiety score was 43.17 (SD = 10.58) (25). This variation might be due to the difference in the Test Anxiety Inventory questions (TAIQ) (the study tool). The previous study utilized twenty 4-point Likert scale statements, whereas the current study utilized ten 5-point Likert scale statements. In addition, the discrepancy might be related to a difference in the teaching-learning environment, such as a curricular difference.

In the present study, students’ current substance use showed a significant association with test anxiety. TA was increased by about 1.03 and 0.12 units among those students who currently used tobacco and chewed khat, respectively. This finding is congruent with a finding from Abuja, Nigeria (26).

Self-esteem was significantly associated with TA. A unit increase in self-esteem decreased the mean score of TA by about 0.05 units. This finding is consistent with previous studies conducted in Turkey (27) and Israel (28).

In the current study, psychological distress showed a significant association with TA. A unit increment in psychological distress increased the mean score of TA by about 0.02. This finding is in line with a finding from the University of Gondar (14).

Moreover, the score of TA decreased by about 0.16 units among students performing regular physical activity daily as compared with students performing no physical activity daily. This finding is congruent with the previous studies that explored how daily physical activity can alleviate TA and enhance test performance (29–31).

### Limitation and strength of the study

Unlike the previous studies conducted in Ethiopia, this study identified a linear relationship between the independent variables and TA. The study utilized standardized tools to test TA and the psychosocial status of the students, such as psychological distress, social support, and self-esteem. However, it assessed TA at a point in time, which could be its shortcoming. So it is better to conduct follow-up studies in the area in the future. In addition, there might be intra-personal attributes and institutional cultures that could enhance or alleviate TA but were not explored in this study. Therefore, future investigators need to consider a qualitative study in the same population.

## Conclusion

TA was common in the study area. The study identified current substance use (tobacco use and khat chewing) and psychological distress as enhancing factors for TA. On the contrary, self-esteem and physical activity were discovered to be anxiety-relieving factors. Thus, we recommend that the students and all stakeholders, including student guidance and counseling units, university and college administrators, instructors, and the students’ families, work on discouraging those factors that increase test anxiety while promoting the alleviating factors.

## Data availability statement

The original contributions presented in the study are included in the article/Supplementary material, further inquiries can be directed to the corresponding author.

## Ethics statement

The study followed the principles of the Declaration of Helsinki (32). First, an ethical approval letter was obtained from the Institutional Research Ethics Review Committee (IRERC) of the Health Sciences College, Mattu University. Then a letter of cooperation was submitted to each department. After a detailed explanation of the study’s purposes and risks, each participant signed an informed consent form willingly. They were free to leave at any time during the interview. In addition, the names or identifiers of the participants were not mentioned in the data.

## Author contributions

MJ conceived and validated the study, proposed methods, planned software, collected resources, administered the study, participated in the statistical analysis, and wrote up the manuscript. WG conceived the study, proposed methods, participated in the statistical analysis, manuscript draft write-up, and revision of the final manuscript. MT, AM, and TT collected resources, supervised the study process, and participated in the statistical analysis and manuscript write-up. All authors contributed to the article and approved the submitted version.
